# Red Cross Accountancy: The Policy of the Big Balance

**Published:** 1917-03-10

**Authors:** 


### RED CROSS ACCOUNTANCY.

### The Policy of the Big Balance.

The Report of the Joint Finance Committee of the British Red Cross Society and the Order of St. John of Jerusalem, which has now been issued, covers the accounts for the first two years of the war. There are one hundred and twenty-four pages in which the balance-sheet, the schedules of votes and grants, the details of the expenditure on certain units, the register of ambulances, boats, and stores, and, finally, a batch of statistics are marshalled in succession. Up to date there has been a sum of practically six millions to spend, but by October 20 last, the end of the period which is covered by the Report, the total received was £4,977,227.

To understand the position at a glance a table is necessary.

| Total Income | | Total Expenditure | |
|---|---|---|---|
| 1915 ... | £1,912,995 | 1915 ... | £1,691,230 |
| 1916 ... | 3,064,232 | 1916 ... | 1,815,353 |

Total Balance in Hand.

| | |
|---|---|
| On October 1916 ... ... | £1,470,644 |
| Our Day Collection, over ... | £1,000,000 |

The outstanding point in the Report, therefore, is that the Joint Committee has in hand to-day considerably more than the sum total at which its expenditure for the year has been estimated. That is a sufficiently remarkable fact. What does the committee say about it, and how does it justify a continued attitude of appeal? The argument is contained in the following passage:—

"While we have spent over £1,800,000 in the past year, we have received a sum so much greater that we have in hand to-day a balance sufficient to meet our estimated expenses during the current year. But as no one can tell us how long the war will last, or how long the present inflow of public generosity will continue, or what may be the amount of our liabilities when peace is declared (and when our revenue almost certainly will cease), reserves such as we carry to-day cannot be regarded as excessive."

### A Policy of Caution.

That is the suggestion, and to indicate how the money, which seems as if it would continue during the war to exceed the expenditure of the fund, may possibly in the end be spent, the committee adds that "there will remain a choice of institutions for the after-care of men broken in the war, to some of which any balances which we may hold could rightly and properly be devoted."

Before considering one or two points in the various sections of the Report it may be well to remark two tendencies to which very large mushroom funds, started to meet a sudden emergency, are subject. The first is naturally to make the most of public generosity, and the second is to set aside and perhaps invest a large part of the total subscriptions. Yet such money was subscribed to be spent, to meet a present need, and, if generously provided, to meet it handsomely. The field of charitable work is dominated by an excessive fear lest anyone should be overpaid from charitable funds, and so we find in hospitals and institutions very slow advances in salaries notwithstanding the admitted fact that hospital staffs are very much underpaid at the present time. Hospital chairmen even have been heard to describe the current salaries of nurses as disgraceful. It is not unreasonable, therefore, to suggest that those responsible for big charitable funds should remember, first, that the money entrusted to them is subscribed to be quickly spent, and secondly, that, once reasonable care has been taken to ensure support for future expenses, the condition of those who carry on the work for the benefit of the patients should not be forgotten. Charities with money in hand are in a position to do their work handsomely. To insist on the grantees making proper provision for salaries and pensions is as much their duty as to encourage a high standard of administration generally. King Edward VII.'s Hospital Fund for London exercises the power of the purse to secure a high general level of efficiency. Why, therefore, should not the British Red Cross Society give a gentle hint now and again to any military hospitals, in this country at least, which are not making proper provision for the comfort of their patients or the remuneration and well-being of their staffs? We make this suggestion since we have some reason to believe that in some of the new military hospitals, especially in those accommodated in temporary buildings, the conditions under which the patients and staff are expected to work or recover fall short of reasonable comfort and efficiency in such matters as food, warmth, and hours of work. In short, our contention is that the policy of the big balance requires two justifications. The first is that offered by the Joint Committee—namely, the need for prudence and a margin for the future. The second, of which too little is heard, is that grants should be accompanied by some form of supervision. Sir W. Plender has pointed out the increasing claims of local funds, but in a complex situation the consideration that we present must also be remembered.

We have space only to comment on one of the funds, whose statistical details necessarily form the bulk of the Report; and the variety of work represented by these statistics has been given such wide publicity that we make no apology for any omissions. Among the funds of the Joint Committee is one known as the "Compassionate Fund (Red Cross *Personnel*)." The fund "exists to meet any proper claims made by those of our staff and *personnel* who are not covered by insurance. A sum of £547 has been given in grants, and the balance of this fund is £22,035. The Joint Committee congratulates itself that the claims on this fund up to date have been small. That is certainly well so long as proper means are taken to bring the fund's existence to the notice of the staff, and to make all grants which are approved as generous as the response of the public amply warrants.

